# Tuberculosis—Learning the Impact of Nutrition (TB LION): protocol for an interventional study to decrease TB risk in household contacts

**DOI:** 10.1186/s12879-021-06734-z

**Published:** 2021-10-12

**Authors:** Chelsie Cintron, Prakash Babu Narasimhan, Lindsey Locks, Senbagavalli Babu, Pranay Sinha, Nonika Rajkumari, Vaishnavi Kaipilyawar, Anurag Bhargava, Kimberly Maloomian, Padma Chandrasekaran, Sheetal Verma, Noyal Joseph, W. Evan Johnson, Christine Wanke, C. Robert Horsburgh, Jerrold J. Ellner, Sonali Sarkar, Padmini Salgame, Subitha Lakshminarayanan, Natasha S. Hochberg

**Affiliations:** 1 Department of Medicine, Section of Infectious Diseases, Boston Medical Center, Boston, MA USA; 2 Department of Preventive and Social Medicine, Jawaharlal Institute of Postgraduate Medical Education and Research, Puducherry, India; 3 Department of Health Sciences, Boston University College of Health and Rehabilitation Sciences Sargent College, Boston, MA USA; 4 Department of Medicine, Center for Emerging Pathogens, Rutgers New Jersey Medical School, Newark, NJ USA; 5 Department of Internal Medicine, Yenepoya Medical College, Mangalore, Karnataka India; 6 Center for Bariatric Surgery, The Miriam Hospital, Providence, RI USA; 7 Department of Clinical Research, National Institute for Research in Tuberculosis, Chennai, India; 8 Division of Computational Biomedicine, Boston University School of Medicine, Boston, MA USA; 9 Department of Public Health & Community Medicine, Tufts University School of Medicine, Boston, MA USA; 10 Department of Epidemiology, Boston University School of Public Health, Boston, MA USA; 11 Department of Medicine, Section of Infectious Diseases, Boston University, School of Medicine, Boston, MA USA

**Keywords:** Latent tuberculosis infection, Undernourished, Parasite infection, Nutritional supplementation

## Abstract

**Background:**

Comorbidities such as undernutrition and parasitic infections are widespread in India and other tuberculosis (TB)-endemic countries. This study examines how these conditions as well as food supplementation and parasite treatment might alter immune responses to *Mycobacterium tuberculosis* (Mtb) infection and risk of progression to TB disease.

**Methods:**

This is a 5-year prospective clinical trial at Jawaharlal Institute of Post Graduate Medical Education and Research in Puducherry, Tamil Nadu, India. We aim to enroll 760 household contacts (HHC) of adults with active TB in order to identify 120 who are followed prospectively for 2 years: Thirty QuantiFERON-TB Gold Plus (QFT-Plus) positive HHCs ≥ 18 years of age in four proposed groups: (1) undernourished (body mass index [BMI] < 18.5 kg/m^2^); (2) participants with a BMI ≥ 18.5 kg/m^2^ who have a parasitic infection (3) undernourished participants with a parasitic infection and (4) controls—participants with BMI ≥ 18.5 kg/m^2^ and without parasitic infection. We assess immune response at baseline and after food supplementation (for participants with BMI < 18.5 kg/m^2^) and parasite treatment (for participants with parasites). Detailed nutritional assessments, anthropometry, and parasite testing through polymerase chain reaction (PCR) and microscopy are performed. In addition, at serial time points, these samples will be further analyzed using flow cytometry and whole blood transcriptomics to elucidate the immune mechanisms involved in disease progression.

**Conclusions:**

This study will help determine whether undernutrition and parasite infection are associated with gene signatures that predict risk of TB and whether providing nutritional supplementation and/or treating parasitic infections improves immune response towards this infection. This study transcends individual level care and presents the opportunity to benefit the population at large by analyzing factors that affect disease progression potentially reducing the overall burden of people who progress to TB disease.

*Trial registration* ClinicalTrials.gov; NCT03598842; Registered on July 26, 2018; https://clinicaltrials.gov/ct2/show/NCT03598842

**Supplementary Information:**

The online version contains supplementary material available at 10.1186/s12879-021-06734-z.

## Background

Tuberculosis (TB) is one of the most common infectious causes of death worldwide resulting in 1.2 million deaths in 2019 [1]. One-quarter of the world is infected with *Mycobacterium tuberculosis* (Mtb) and 10% of these individuals develop TB over a lifetime [1]. India has a quarter of the global TB burden with 2.64 million cases and 436,000 deaths in 2019 [1]. The Indian government has committed to an 80% TB incidence reduction by 2025 [2]. However, even as the Indian government invests in improved testing, it has not made commensurate efforts in addressing the leading risk factor for the spread of TB in India: undernutrition. This is a critical shortcoming because more than 50% of TB cases in India are attributable to undernutrition [3], and in Puducherry, as many as 61.5% of TB cases in women and 57.4% in men are attributable to undernutrition [4].

Undernutrition is widely prevalent in India—the World Bank reported in 2018 that 14% of India’s total population was undernourished [5] with the National Family Health Survey reporting 22% and 19% of men and women, respectively, with low BMI in 2017 [6]. A recent systematic review found a log-linear relationship between BMI and TB incidence with a 14% decrease in TB incidence with every unit increase of BMI [7]. Furthermore, a longitudinal study in the United States found that having BMI < 18.5 kg/m^2^ is associated with an adjusted hazard ratio of 12.43 for developing TB disease compared to those with BMI ≥ 18.5 kg/m^2^ [8]. In addition, undernutrition is associated with increased risk of TB treatment failure and mortality [9], greater lung involvement in TB disease [10], and risk of drug-associated toxicities despite weight-based dosing [11].

Our understanding of how undernutrition affects the immune response to Mtb is limited. Animal models suggest undernutrition has an impact on innate and adaptive response [9] affecting macrophage mobility, chemotaxis, attachment, and phagocytosis [12]. Human studies of undernourished individuals showed that CD4^+^ and CD8^+^ T-cells needed for an effective T-helper (Th)-1 response may be sequestered in lymph nodes at the expense of the peripheral circulation [13] and that Th1 cytokine responses (needed to control Mtb infection) are decreased in such individuals [14, 15]. Detailed data on these mechanisms are lacking and hence it is not clear how exactly undernutrition impairs the immune responses. Research on the benefits of nutritional supplementation for TB prevention has heretofore primarily focused on vitamin D [16–19] with limited research on macronutrient or other micronutrient supplementation [20].

Undernutrition from inadequate caloric intake due to food insecurity can be exacerbated by parasitic infections that are common in India [21]. Intestinal parasites can decrease absorption of key macronutrients such as proteins and fats as well as micronutrients like zinc and iron [21]. Moreover, animal and human studies have shown that parasites can decrease food intake by inducing gastrointestinal symptoms such as nausea and diarrhea and increasing expression of cytokines like TNF-α and IL6 [7].

The Tuberculosis—Learning the Impact of Nutrition (TB LION) study thus aims to define the specific points at which undernutrition, parasitic infection and Mtb infection intersect, by identifying the mechanisms of the interaction and the response to intervention. These study findings will have relevance in India and other TB-endemic countries worldwide.

## Methods/design

### Aims, objectives, and outcomes

TB LION addresses four aims and outcomes: (1) Examine the effect of undernutrition and parasitic infections on innate and adaptive immune responses, Mtb killing and antigen-specific memory T cell responses in adult household contacts of patients with active TB; (2) Perform whole blood transcriptomics to gain insight into mechanisms of how malnutrition and parasitic infections modulate the immune response to Mtb infection and compare TB risk signatures in these groups; (3) Examine the effect of nutritional supplementation and (4) parasite treatment, on the above immune responses and RNAsequencing signatures.

### Study design and setting

TB LION is a prospective, exploratory clinical trial conducted at the Jawaharlal Institute of Postgraduate Medical Education and Research (JIPMER) in partnership with Boston Medical Center/Boston University and Rutgers New Jersey Medical School. JIPMER is located in Puducherry, India, a city of approximately 1.2 million [22]. TB LION enrolls participants across Puducherry and two districts of Tamil Nadu: Cuddalore (population ~ 2.6 million), and Villapuram (population ~ 3.5 million). Three field teams comprised of trained social works and nurses are responsible for study enrollment and participant follow up. Enrollment began in Puducherry in July 2019.

### Study population

Study staff identify new TB index cases (ICs) through the National Tuberculosis Elimination Programme (NTEP). Inclusion criteria are: (1) Age ≥ 18 years; (2) New sputum-smear positive (≥ 1+ acid fast bacilli [AFB]) pulmonary TB that is culture or GeneXpert positive; (3) No history of TB treatment; (4) Have at least one household contact (HHC) with whom they shared a house during the previous month; (5) Agree to have HHCs contacted. Exclusion criteria include: (1) Pregnant; (2) No GeneXpert or culture confirmation and unable to provide sputum sample; (3) No HHCs; (4) Known MDR or XDR TB; (5) BMI < 14 kg/m^2^ as to avoid refeeding complications; (6) Kwashiorkor, lower extremity edema or lower extremity neuropathy in those with BMI < 14.

Inclusion criteria for HHC include: (1) Age 18–60 years; (2) Housemate of an eligible TB case for at least the past month; (3) HIV seronegative or willing to be tested; (4) Willing to be tested for pregnancy. Exclusion criteria include: (1) Pregnant at enrollment; (2) Having known diabetes mellitus or evidence by hemoglobin A1C (HbA1c) > 6.5%; (3) GeneXpert positive among those able to produce sputum; (4) History of TB disease; (5) Karnofsky score ≤ 10 (moribund); (6) If BMI < 16 kg/m^2^, abnormal potassium, magnesium, or phosphorus; (7) Kwashiorkor; pitting edema; neuropathy [23].

All HHCs in the home are approached for enrollment. All eligible household contacts in one house are enrolled for prospective follow up if the HHCs fall in overlapping study arms. If there are more than one eligible HHCs, but no overlapping study arm designations, any malnourished HHC screened is enrolled to limit selection bias. We expect to enroll 760 HHCs to identify 120 study participants for longitudinal follow-up. Sample size was determined by participant data from the Regional Prospective Observational Research for Tuberculosis (RePORT India) cohort study [24].

### Study procedures

#### Index case and household contact enrollment

**Fig. 1:**
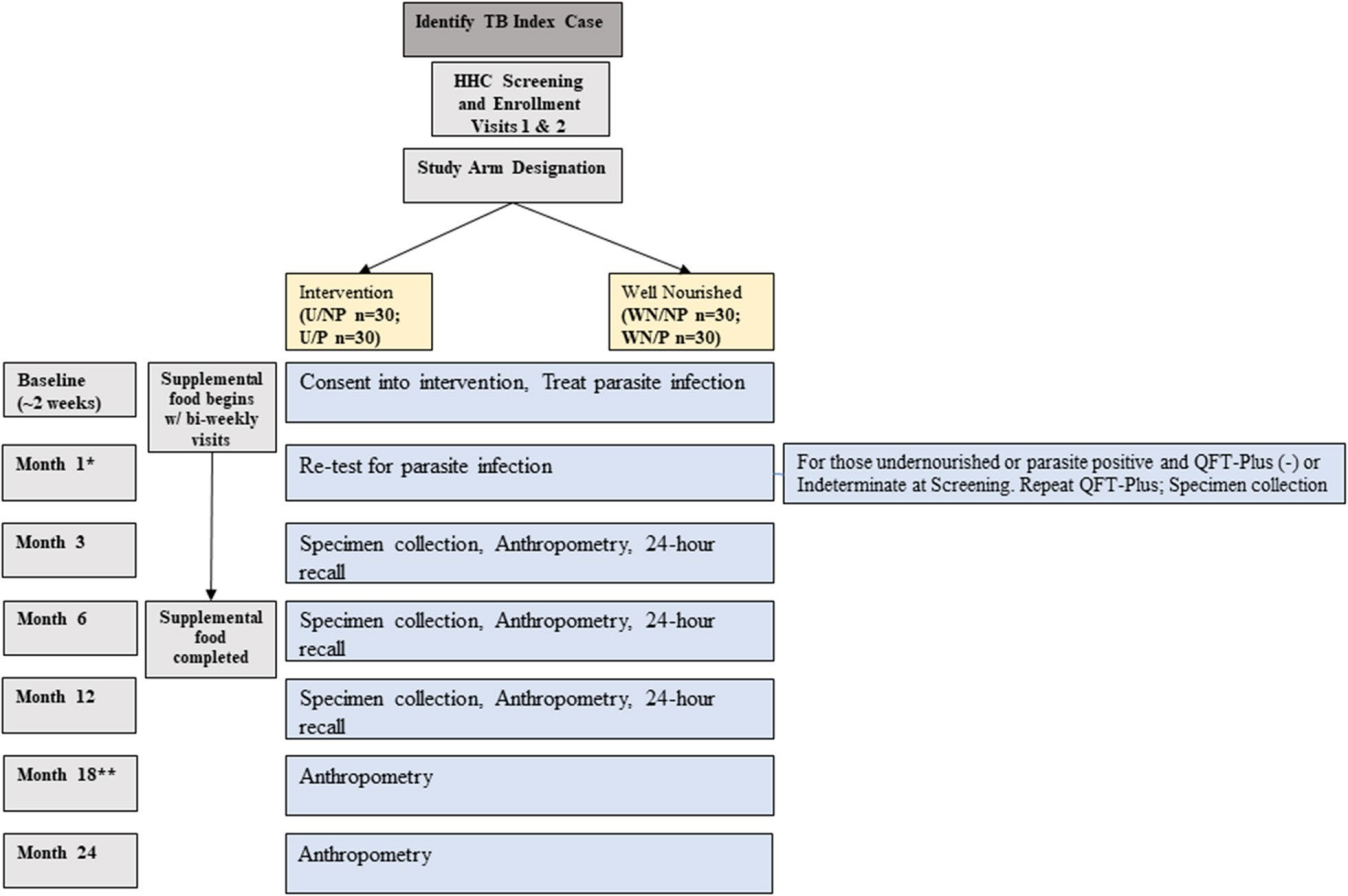
Tuberculosis: Learning the Impact of Nutrition (TB LION) study schematic. *For parasite participants only, **For intervention participants only. *U* Undernourished, *WN* Well-nourished, *P* Parasite-infected

Participants are enrolled and consented at their primary healthcare center (PHC), hospital, or other place of their choosing. Laboratory evaluation of sputum for GeneXpert or AFB smear/mycobacterial culture, chest x-ray (CXR) and urine pregnancy test is conducted to determine eligibility status for ICs. Medical history, medication history, and BMI will also be collected. Index cases are not followed after that initial visit, unless the IC and enrolled HHC is malnourished then the IC will receive up to five household visits. HHCs are contacted and evaluated for study eligibility and participation 8 weeks after IC enrollment (Fig. 1). Evaluation will take place at their PHC, home, or other place of their choosing. Study staff obtain written informed consent for all ICs and HHCs. Additional consent is obtained to use stored samples for future research.

### HHC participant assessments

#### Demographics and medical history

**Fig. 2:**
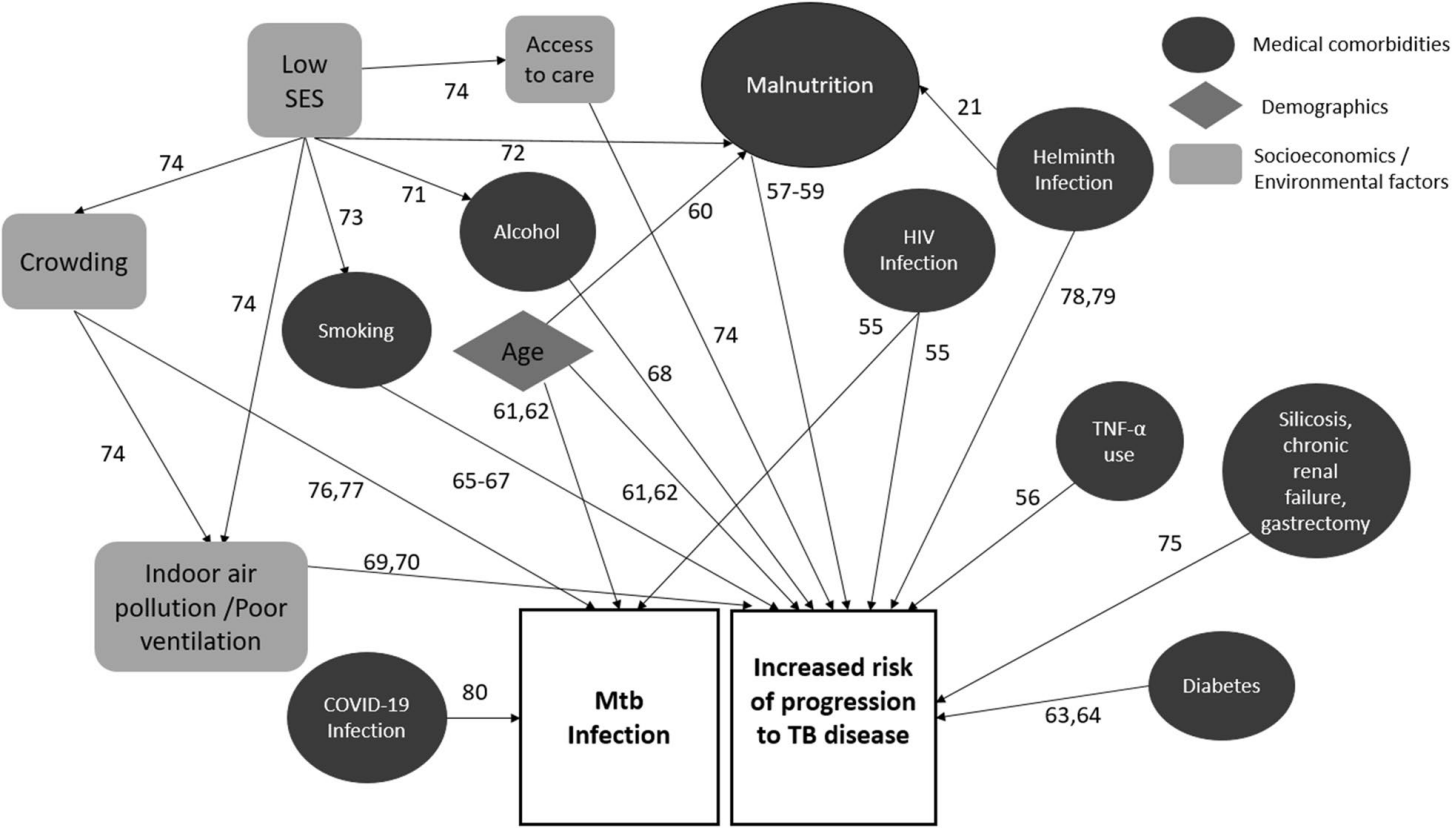
Schematic of factors associated with *Mycobacterium tuberculosis* (Mtb) infection and risk of progression from Mtb infection to active TB disease with numbered references [55–80]

Participants have demographic information collected at baseline and during follow up visits (months 3, 6, 12, 18 and 24). Information regarding socioeconomic status [25, 26] (Multidimensional Poverty Index and the Demographics and Health Surveys Program), alcohol consumption [27] (Alcohol Use Disorders Identification Test-Concise [AUDIT-C]), smoking history [28] (WHO Global Adult Tobacco Survey), household food insecurity [29] (USAID Household Food Insecurity Assessment Survey), quality of life [30] (36-Item Short Form Survey Instrument), and stigma [31] (Courtesy Stigma Survey) are collected as these factors may be associated with Mtb infection (Fig. 2). Participants are screened for TB symptoms at baseline and during follow up visits (bi-weekly, months 3, 6, 12, 18 and 24).

### Nutritional assessments

#### Anthropometry

Participants undergo anthropometric assessment (height, weight, mid-upper arm circumference, hip circumference, waist circumference, triceps and subscapular skinfold thickness, and grip strength) at baseline and during follow up visits (months 3, 6, 12, 18 and 24) [32, 33]. All measurements are taken in triplicate. Field teams have comprehensive anthropometric training; a trained dietitian from the US has conducted a week-long training, introduced each measurement tool and demonstrated taking each measurement to the site staff. Each staff member was required to pass a practical examination prior to study start and at regular intervals thereafter. Videos were made of the training sessions and are viewed at 6-month intervals by the study team.

#### Assessment of dietary intake

At enrollment/screening and follow-up visits (months 3, 6, 12, and 24), nutritionists conduct detailed 24-hfood recalls to assess dietary intake [34]. A 5-step multiple pass method, based on the USDA method, is used [35]. In step one, participants recall an uninterrupted list of all food and drink items consumed in the last 24 h probing for any forgotten items. Each item is entered into the data collection instrument, and the nutritionist reviews each list item again and collect data on portion size or quantity. The next three steps use a structured approach to collect details on preparation methods and portion sizes. Nutritionists were trained to use portion-size guide props including locally used dishes, cups, and serving utensils to serve as a visual aid for participants. Each standardized item is labeled with the appropriate milliliter or gram size, and participants are encouraged to report whether they consumed the whole or partial amounts of portions. The final step probes for forgotten foods and includes several additional memory cues.

Nutritionists collect 24-h recalls on paper forms before entering the data into a tailor-made, local food composition database housed in DietSoft (New Delhi, India). DietSoft software contains 1205 commonly consumed Indian food items including all of the foods in the India Food Composition Tables (IFCT) [36] and the National Institute of Nutrition (NIN) Nutritive Value of Indian Foods list [37], as well as several popular brand-name items. The study team also conducts extensive recipe collection in order to supplement DietSoft with common recipes relevant to the diets of people in Tamil Nadu, specifically. Recipes are collected from study participants, relevant household members, or from community members responsible for cooking (restaurant/shopkeepers and study nutritionists). When possible, recipes are collected using a direct observation method that includes measuring the weight and volume of all raw ingredients, recording details on preparation methods (e.g., frying, boiling, etc.), and measuring the weight and volume of the cooked dish. When direct observation is not possible, nutritionists collect recipes from respondents using a multiple-step method similar to the USDA 24-h recall data collection methodology [35]. For recipes with wide varieties in preparation methods, they are collected from multiple respondents to capture variability. Study nutritionists supplement the DietSoft food item list with nutrition information from commonly consumed brand-name items that were not pre-entered in DietSoft.

### Parasitology

Participants are provided with stool containers for at home stool collection during screening and follow up visits (months 1, 3, 6, and 12). Stool samples are collected in three containers containing (1) 10% formalin (for diagnostic microscopy); (2) Zn-PVA (for polymerase chain reaction [PCR] testing); and (3) RNAlater (for microbiome testing on a subset of participants). If there is a shortage of Zn-PVA, stool samples are split into two with half the sample being stored in 5–10% formalin and the other half unpreserved. QuantiFERON-TB Gold Plus (QFT-Plus) negative screened participants only have stool collected for microscopy. PCR is conducted to confirm infections with *Ancylostoma duodenale, Necator americanus, Ascaris lumbricoides, Strongyloides stercoralis*, and *Trichuris trichiura*. We define parasite infection as having one or more helminth infections. Those with helminth infections are referred to their PHC for treatment. Month 1 microscopy and PCR is conducted on QFT-Plus positive parasite study arm participants to confirm that parasite infections have resolved. The parasitology technician conducts stool concentration, sedimentation, and trichrome staining for parasite identification. All parasite positive samples and diagnostic queries undergo confirmation by the head of the parasitology department. The parasitology technician completes a quarterly external quality assurance (EQA) program (Wisconsin State Laboratory of Hygiene) [38]. All remaining samples are stored in – 80 C freezer.

### Longitudinal follow-up

HHCs that are QFT-Plus positive are placed in one of four study arms for longitudinal follow-up over 2 years (30 in each): (1) Undernourished (BMI < 18.5 kg/m^2^) with parasitic infection (UP); (2) Undernourished without parasitic infection (U); (3) Well-nourished with parasitic infection (WP) and (4) Well-nourished without parasitic infection (W). The field team will attempt to enroll participants evenly across all study arms until target numbers in each arm are met. If enrollment challenges are presented, we will extend the enrollment period by 1 year. Study staff make all attempts to retain each participant in the study via training in rapport building and behavior change as well as having frequent contact through study visits. However, participants are free to discontinue participation at any point.

Participants are evaluated at 7 additional time points after enrollment (2 weeks and months 1, 3, 6, 12, 18 and 24) to monitor change in health status and evaluate change in immune response. All HHCs are screened for TB symptoms at every visit. If a HHC develops TB symptoms at any visit, sputum, blood, and stool samples are collected and the HHC is referred to NTEP for evaluation. Participants who cannot be reached after three contact attempts (on varying days and times) are moved off study and considered lost to follow up. Case report forms assess potential adverse reactions to the intervention, and study staff are trained to report any adverse event to the Principle Investigators (PIs) who determine the correct reporting path to their respective review boards.

### Two-month repeat QFT-plus

For any malnourished or parasite positive participant with a negative or indeterminate QFT-Plus result during screening, a follow up visit is conducted approximately 2 months later for repeat QFT-Plus. Any HHC positive upon repeat testing is enrolled for longitudinal follow up. For those who are malnourished, still QFT-Plus negative upon repeat testing, but have a HHC already enrolled into the intervention due to malnutrition, the participant is enrolled into the study for longitudinal follow up.

### Nutritional intervention

Undernourished participants are re-consented and enrolled into the study intervention (food and micronutrient supplementation) for 6 months followed by an additional 18-months of extended follow-up. In order to encourage adequate nutritional intake, even in the context of food sharing, the nutrition intervention food rations provided are sufficient to feed all household members. At enrollment, participants are asked the number and age of household members.

The kcal requirements for the food supplements were derived from the Indian TB Nutrition Guidelines that men and women require 2500 and 2100 kilocalories (kcal) respectively; thus, HHCs receive 2600 kcal each with children 13 years and younger receiving 1300 kcal [39]. The 2600 kcal/day needs were determined based off clinical feeding guidelines of 35–40 kcal/kg for undernourished patients [40] to achieve a BMI level considered optimal (21 kg/m^2^) based on the National Institute of Nutrition (NIN) in India’s recommendation for optimizing health and nutrition. In order to determine the contents of the food ration, we consulted local partners, TB experts in India, and nutritionists to create a meal package that is locally acceptable, suitable for vegan and omnivores, and that gives participants dietary variety without compromising nutritional quality. The meal package is comprised of pulses, white rice, groundnuts, and oil; nutrition calculations were made from standard nutrient analysis based on the *Exchange Lists for Meal Planning*, by the Academy of Nutrition & Dietetics in conjunction with the American Diabetes Association [41] (Table 1). These foods were also selected because they can be easily transported to the participant homes and they do not require refrigeration. Enrolled participants also receive a multiple micronutrient supplement (Life-Extension Two-Per-Day Tablets) [42] which contains over 100% of the RDA for Vitamins A, B6, B12, C, D3, and E.

**Table 1:** Tuberculosis: Learning the Impact of Nutrition (TB LION) nutritional intervention package for one adult per day

Food category	Food item	Kilocalories	Protein (g)	Fat (g)
Fat (5 g/day)	Vegetable oil	45	0	5
Pulse (117 g/day)	Red gram, green gram, and black gram	468	23.4	1.5
Grains (325 g/day)	White rice	1301	22.75	2.3
Nuts (143 g/day)	Ground nuts	786	35.75	70.1
Total		2600	81.9	78.8

The field team visits households every other week to replenish the food, assess food consumption and changes in participant health. During the initial food delivery visit, nutritionists provide study food containers and then weigh each container, taring the scale to accurately account for the weight of container. They then add the calculated amount of each food item and mark each container with a black marker to indicate the fill line. The weight of each respective food container is recorded. Nutritionists return to the household every 2 weeks to evaluate the remaining food and vitamin supplement. The remaining food (for each item) is poured into a tared tray, weighed and recorded. Using the initial calculated food quantity and remaining quantity, nutritionists calculate the amount of food consumed by the household and the amount required to replenish the stock which is added to the container before the remaining food to minimize food expiry.

Severely undernourished participants (BMI 14–16) receive partial supplementation for the first 2 weeks of intervention; 30% for the first week and 50% the second week. By the third week, participants will receive 100% of the supplementation. Study staff assess electrolytes (potassium, magnesium, and phosphorus) and conduct an assessment for food consumption and changes in participant health before replenishing the week’s food supply. If electrolyte results are normal, food supply is increased; however, if food is not being consumed based on observation, a second week of the partial ration is provided and electrolytes re-checked. If electrolyte results are abnormal, food supplementation is stopped, and the participant is referred to the hospital. HHC bi-weekly visits detailed above will begin once 100% of the ration is received.

### Data management

Data, for those screened and enrolled, is entered into paper case report forms that are reviewed in person for completion by the study research officer. Each form is manually reviewed for completion and quality control. Incomplete forms are returned to the field team for rectification. Field teams then upload all data into the Research Electronic Data Capture (REDCap), a secure, web-based software platform designed to support research studies, providing an intuitive interface for validated data capture, built-in quality control and assurance checks, and audit trails for tracking data (Vanderbilt University, USA) [43, 44].

Data on the server is verified by reviewing electronic data against paper copies by a designated staff member. The verification process is subsequently used to check that provided responses are valid. Inconsistencies or incomplete forms are returned to the field team for rectification. The study coordinator in Boston will review all data for completion once on the REDCap server and will maintain a query log that is routinely sent to the field team for rectification. Regular data reviews are generated on the REDCap platform and in Statistical Analysis Software (SAS, Cary, NC, USA). All onsite study activity is monitored by the site PIs and study coordinator. Weekly meetings are held to address and rectify any issues that arose during the week.

Any de-identified data and statistical code used for analyses may be made public if required for manuscript publication. The study PIs will evaluate requests and give access to the data for analysis and publication of study results. All PIs and authors will be responsible for reviewing and approving any manuscript before publication.

### Specimen handling and storage

Plasma, serum and whole blood are collected serially over 1 year (Additional file 1: Table S1). Testing is performed for complete blood count (CBC), albumin, C reactive protein (CRP), HIV, and Hemoglobin A1c; for severely malnourished IC and HHC, electrolytes (potassium, magnesium, and phosphorus) are measured. Additional serum samples are aliquoted and frozen. QFT-Plus (QIAGEN, Germany) testing is performed and analyzed as per package instructions; supernatant is extracted and stored at − 80 °C. Tuberculin skin tests (TST; ARKRAY, USA) is administered upon screening and repeated at six months if initial TST was negative (< 5 mm) [45]. Whole blood is collected in PAXgene tubes for RNA extraction and stored at − 80 °C. For isolation of PBMCs, whole blood is collected in Sodium Heparin and K2 EDTA (BD Biosciences) tubes. Plasma is harvested prior to whole blood dilution for PMBC processing. Blood is diluted in PBS and processed using Ficoll-Plaque density gradient media to isolate PBMCs. Following cell count, 5–10 × 10^6^ PBMCs is aliquoted into cryovials in fetal bovine serum (FBS) containing 10% dimethyl sulfoxide (DMSO), stored overnight at − 80 °C and then transferred to liquid nitrogen for long-term storage. The remaining plasma is stored at − 80 °C. Stool samples are collected (as described above). Plasma and serum is collected in K2 EDTA-10.8 mg tubes, wrapped in aluminum foil for light shielding, and stored at − 80 °C for future micronutrient testing. Sputum samples are processed (as described above) and stored in cryo-containers at − 80 °C. Urine samples are assessed for pregnancy using urine spot tests. Field and laboratory staff are trained in and follow strict sample collection, transportation and storage protocols.

### Assessment of intracellular bacterial growth in Mtb-infected macrophages

After banking a sufficient number of specimens, we will begin the detailed immunologic assessments. We will use a rapid and high-throughput Mtb-luciferase based assay in peripheral blood monocyte (PBMC)-derived macrophages to examine the effect of undernutrition and parasitic infections on Mtb growth restriction in vitro. This assay measures Mtb growth in macrophages using recombinant Mtb expressing the whole bacterial lux operon (luxCDABE). This assay will produce measurements in the form of relative luminescence units. PBMC will be infected with Mtb at a multiplicity of infection (MOI) of 5 for a 7-day growth assay. Kinetics of luminescence generated will be monitored daily using a luminometer plate reader. Reading at day 0 and daily measurements of luminescence in the same well will allow for correction of minor variations in the number of input cells from each sample when calculating percent growth of Mtb in macrophages.

### Evaluation of antigen-specific memory T-cells

Several subsets of memory T cells exist, including stem cell memory (T-SCM), central memory (T-CM), transitional memory (T-TM) and effector memory (T-EM). Development of memory CD4^+^ T-cells is critical for protection against TB disease [46] and yet, the impact of undernutrition and parasitic infections on the responsiveness of Mtb antigen-specific T-cells has not been fully explored. In this study, we will characterize Mtb antigen-specific memory T-cell phenotypes and functionality, to broaden understanding of the impact of undernutrition and parasitic infections on these cells, beyond that of IFNγ expression. In addition, the evolving memory T-cell response in undernourished individuals, with and without feeding, will also be examined. We will use multiparametric flow cytometry and use cell surface markers to compare memory T-cell subsets in PBMCs collected from the different groups. Freshly thawed, cryopreserved PBMCs will be stimulated with ESAT-6, CFP-10, and Ag85B in the presence of anti-CD28 and anti-CD49d antibodies for 16 h. To mark the antigen specificity of memory subsets, expression of IFNγ, IL-2, TNFα and IL-17 will be evaluated by intracytoplasmic staining. Expression of PD1, a marker of T cell exhaustion, will be examined in each memory T cell subset. The FLOW cytometry staining panel also includes antibodies reactive against FoxP3 and CD25 to identify Tregulatory (Treg) cells that impact CD4^+^ T-cell proliferation and function.

FLOW Jo software (Tree Star Inc., San Carlos, CA) will be used to analyze the data to obtain frequency of T-SCM, T-CM, T-TM and T-EM cells. To calculate antigen specific T cells within each of the T cell memory subset, we will determine the percentage of cytokine positive cells (single, double and triple producers are grouped together as cytokine positive cells).

#### Sample size calculations

##### RNA-sequencing

We have two studies validating existing (or newly derived) biomarkers in independent cohorts—distinguishing TB vs. latent TB infection (LTBI) (n = 28 and n = 16, respectively) and predicting progression from LTBI to TB disease (n = 16 progressors and n = 11 controls) [47, 48]. Both studies demonstrated that all biomarkers had AUCs > 0.80 and p-values < 0.0001; therefore, our study with n = 30 samples per group is more than sufficient to apply and validate existing biomarkers.

##### Novel biomarker development

We re-analyzed expression data from a large transcriptomic study (n = 491); there are significant gains in sensitivity when increasing from 10 to 30 samples within groups, and only a moderate benefit of increasing from 30 to 40 in each group [49]. Specificity requires fewer samples, as it stops improving significantly after 20 samples per condition. Thus, these results justify our sample size of 30 per study arm.

##### Immunologic analyses

In our preliminary work, we used a bootstrap approach to estimate the expected estimation error for the AUC, sensitivity, and specificity for our T cell markers at our proposed training sample size (n = 30). We expected to be able to estimate the AUC within 0.10, the sensitivity within 0.17, and the specificity within 0.13 using single markers alone. We fully expect that combination biomarkers will lead to more accurate estimates (the combination marker accuracy cannot be estimated because our preliminary data lead to perfect predictions). However, the single marker predictions demonstrate that our sample size is large enough to estimate these quantities with sufficient accuracy.

### Statistical analyses

#### Mtb killing, cytokine analyses, and cellular immune responses

For the Mtb killing (intracellular bacterial growth) assays, we will log-transform relative luminescence units and compare groups using ANOVA including nutrition status, parasite status, gender and any other significant covariate using a forward selection approach. For all analyses, if needed (e.g. skewed or bimodal data), we will apply a non-parametric Kruskal–Wallis test. For colony forming unit measurements, we will use a Poisson regression model that includes covariates using a forward selection approach. For cytokine analyses, markers will be compared using ANOVA or Kruskal–Wallis test. If needed, to correct for multiple comparisons, we will apply a Bonferroni adjustment. To understand the effect of parasite treatment and nutritional supplementation over time, we will use time-course repeated measurements on the same individual and a mixed linear model.

The predictive value will be evaluated by receiver operating characteristics (ROC) curve analysis and logistic regression modeling using covariate metadata. Because our preliminary data demonstrate a strong signal from these markers, we will split our cohort into a training set (n = 20 in each group) and a test set (n = 10 in each group) for cross-validation purposes. The markers will be evaluated for their predictive abilities individually, as well as in combination. The combination logistic regression biomarker will be selected using a forward-step approach, adding markers one at a time and evaluating the predictive value (cross-validation within the training set).

#### RNA sequencing

RNA will be extracted from our samples and sequencing libraries will be prepared and sequenced on an Illumina sequencer as described in our previous work [50, 51]. We will perform transcriptomic analysis to understand mechanisms of immune modulation by undernutrition and parasitic infections. Data will undergo quality control, aligned to the most recent human reference genome (hg38) using RSubread, and feature counts will be estimated for each gene as in our previous work [50, 51]. We will use the edgeR package to determine differential gene expression based on a false discovery rate cutoff of 0.05. The data will be subjected to the following: (i) Transcriptional modular/network analysis; (ii) GSVA and ssGSEA scoring of multiple existing TB-related gene expression pathways using our TBSignature profiler (https://github.com/compbiomed/TBSignatureProfiler); and (iii) Ingenuity pathway analysis [51]. We will use the differentially expressed gene sets and/or existing biomarker genes to derive a logistic regression model to develop biomarkers of Mtb infection in undernourished HHC and see if these predict TB risk. To address response to nutritional supplementation and parasite treatment, we will apply a mixed model using the dream R package [52] to account for the repeated measures analysis. We will use an FDR threshold of 0.05 to control for multiple comparisons.

#### Participant data

Participant data pertaining to demographics, socioeconomic status, food insecurity, anthropometry, and body composition will be analyzed through multivariate regression models. All models will be adjusted for independent variables and confounders to assess relative risk. Cross group comparisons will be made through ANOVA analyses.

### Study expectations

In this study, we hypothesize that in HHC of adults with active TB, undernutrition and parasitic infection increase the risk of progression to TB disease and alter the immune response. Further, we hypothesize that nutritional supplementation and treating parasitic infections reverse their effect on the immune response. We anticipate observing that undernutrition and parasitic infection are independently and jointly associated with reduced Mtb killing, increased Th2 and Treg response, and TB risk signatures. We expect that nutritional supplementation improves Mtb killing, increase Th1 response, and alters TB risk signatures. Lastly, we anticipate that treating parasitic infection results in the normalization of immune parameters and the resolution of TB risk signature risk.

## Discussion

The TB LION study allows us to assess an overlooked obstacle for TB elimination in India—undernutrition—and to evaluate the potential role for parasitic infections in the Indian context. There is a paucity of studies that have assessed the impact of undernutrition on the immune response during Mtb infection and more importantly whether these can be reversed with interventions. The TB LION study, with its ample macronutrient and micronutrient supplementation, rigorous monitoring of nutritional intake, and detailed immunologic analyses address these barriers.

One strength of the study is that we address how the impact of undernutrition is modulated by parasitic infections that are common in India. Intestinal parasites can decrease absorption of key macronutrients (e.g., protein) and micronutrients (e.g., zinc) [21]. Parasites may also have a direct effect on risk of TB progression by inducing Th2 responses and weakening Th-1 (and Th17) responses needed to control Mtb [53]*.* Antihelminth treatment in persons with Mtb infection and Schistosoma or Strongyloides coinfection is associated with increased CD4^+^ IFNγ^+^  cells, suggesting improvement in immune response [54], but data are needed to address whether the implications are similar for other parasites. The TB LION study further informs the role of parasitic infection on risk of progression to TB disease, the time course of improvement in these responses after parasite treatment, and the durability of the response. Furthermore, we are able to address the interplay of undernutrition and parasite infections.

The TB LION study is an ambitious study, with potential to yield very meaningful information. We are confident that our design and methodology, with the addition of sample collection to address the impact of potential COVID-19 infection (through serology), provides comprehensive data to draw sound conclusions regarding the risk of progression to active TB among HHCs. Another key strength of this study is the prospective follow up with clinical assessment on all study participants. Extensive data collection on all participants allows for thorough comparative analyses within and across participant groups. We perform a qualitative assessment of the intervention, a cost-effectiveness analysis of nutritional interventions, and assessments of micronutrient contributions to immune response.

The nutritional intervention is a key strength of this study. We have designed an intervention that surpasses dietary requirements for macronutrients while also supplementing micronutrients providing a more complete nutritional supplement for our intervention participants. Our rigorous collection of 24-h recalls on every participant further strengthens comparative nutritional analyses across participant study arms over time.

This study does have potential weaknesses. The samples size within each study arm is limited; however, the study sample size is sufficiently powered to draw immunologic and RNAsequencing conclusions from the data. Another possible challenge lies within the intervention. We are aware that participants may not consume the supplied food as intended. We anticipated this possibility and have incorporated bi-weekly counselling by nutritionists each time the food is delivered. The nutritionists review the amount of food each household consumes, the participant anthropometrics and clinical assessments, and then interview participants to determine barriers to consuming the provided food. The nutritionists have also been trained in behavior change strategies to promote consumption of the supplied foods.

## Conclusion

TB LION study is novel in that we have integrated epidemiologic, nutritional immunologic and bioinformatics methodologies to assess not only the individual effects of undernutrition and parasitic infection on latent TB infection and risk of progression to TB disease, but also the combined effect of these conditions. This study transcends individual level care and presents the opportunity to benefit the population at large by analyzing factors that affect disease progression potentially reducing the overall burden of people who progress to TB disease.

## Supplementary Information

**Additional file 1: Table S1.** Specimen collection schedule for Tuberculosis: Learning the Impact of Nutrition (TB LION) household contact participants.

## Data Availability

Data sharing is not applicable to this article as it does not contain any data.

## Abbreviations

AFB: Acid fast bacilli
AUDIT-C: Alcohol Use Disorders Identification Test-Concise
luxCDABE: Bacterial lux operon
BMI: Body mass index
CRP: C reactive protein
T-CM: Central memory
CXR: Chest x-ray
CBC: Complete blood count
DMSO: Dimethyl sulfoxide
T-EM: Effector memory
EQA: External quality assurance
FBS: Fetal bovine serum
HbA1c: Hemoglobin A1C
HHC: Household contact
hg38: Human reference genome
IC: Index case
IFCT: India Food Composition Tables
JIPMER: Jawaharlal Institute of Postgraduate Medical Education and Research
LTBI: Latent TB infection
MOI: Multiplicity of infection
Mtb: *Mycobacterium tuberculosis*
NIN: National Institute of Nutrition
NTEP: National Tuberculosis Elimination Programme
PBMC: Peripheral blood monocyte
PCR: Polymerase chain reaction
PHC: Primary healthcare center
PIs: Principle Investigators
QFT-Plus: QuantiFERON-TB Gold Plus
ROC: Receiver operating characteristics
RePORT: Regional Prospective Observational Research for Tuberculosis
REDCap: Research Electronic Data Capture
SAS: Statistical Analysis Software
T-SCM: Stem cell memory
Th: T-helper
Treg: Tregulatory cells
TB LION: The Tuberculosis—Learning the Impact of Nutrition
T-TM: Transitional memory
TB: Tuberculosis
TST: Tuberculin skin tests
UP: Undernourished with parasitic infection
U: Undernourished without parasitic infection
WP: Well-nourished with parasitic infection
W: Well-nourished without parasitic infection
