# Supplementary material for: Tuberculosis—Learning the Impact of Nutrition (TB LION): protocol for an interventional study to decrease TB risk in household contacts
Source: BMC Infect Dis. 2021 Oct 12;21:1058. doi: 10.1186/s12879-021-06734-z (PMC8506078; doi:10.1186/s12879-021-06734-z)
Supplement: Supplementary file 1 — Additional file 1: Table S1. Specimen collection schedule for Tuberculosis: Learning the Impact of Nutrition (TB LION) household contact participants. [file 12879_2021_6734_MOESM1_ESM.docx]

Table S1. Specimen collection schedule for Tuberculosis: Learning the Impact of Nutrition (TB LION) household contact participants

| Specimen | Time Point | | | | | | |
| --- | --- | --- | --- | --- | --- | --- | --- |
|  | Screening 1 | ~2 weeks | ~ 1 month | ~ 3 months | ~ 6 months | ~ 12 months | Initial two weeks of intervention  visits |
| Chest x-ray | X |  |  |  |  |  |  |
| QFT-Plus | X |  | X* |  | X | X |  |
| PaxGene | X |  | X* | X | X | X |  |
| PBMC | X |  | X* | X | X | X |  |
| Serum | X |  | X* | X | X | X | X^α^ |
| COVID-19 antibody | X |  | X* | X | X | X |  |
| HIV | X |  |  |  |  |  |  |
| HbA1c | X |  |  |  | X |  |  |
| Whole Blood | X |  |  |  |  |  |  |
| Albumin |  | X |  | X^β^ | X^β^ |  |  |
| CBC |  | X |  |  | X |  |  |
| Sputum |  | X |  |  |  |  |  |
| Stool |  | X | X^┼^ | X^β^ | X | X |  |
| TST |  | X |  |  | X^§^ |  |  |

*For those undernourished or parasite-infected who were QFT-Plus negative or indeterminate; ^┼^ For participants with parasite infection; ^β^For undernourished participants; ^§^For those TST negative; ^α^ For severely malnourished (BMI 14-16) CBC=Complete blood count; HIV = Human Immunodeficiency Virus; HA1C = Hemoglobin A1C; PBMC = Peripheral blood mononuclear cell; QFT-Plus = QuantiFERON-TB Gold Plus; TST= Tuberculin Skin Test
